# The key role of the right posterior fusiform gyrus in music reading: an electrical neuroimaging study on 90 readers

**DOI:** 10.3389/fcogn.2024.1323220

**Published:** 2024-01-17

**Authors:** Alice Mado Proverbio, Giulia Arcuri, Marta Maria Pantaleo, Alberto Zani, Mirella Manfredi

**Affiliations:** ^1^Cognitive Electrophysiology Lab, Department of Psychology, University of Milano-Bicocca, Milan, Italy; ^2^NeuroMI, Milan Center for Neuroscience, Milan, Italy; ^3^School of Psychology, Vita-Salute San Raffaele University, Milan, Italy; ^4^Department of Psychology, Psychologisches Institut, University of Zurich, Zürich, Switzerland

**Keywords:** fusiform gyrus (FG), VWFA, reading, language, music, N170, alexia, dyslexia

## Abstract

**Introduction:**

In this study, we employed a combined electromagnetic recording technique, i.e., electroencephalogram (EEG)/event-related potentials (ERPs) plus *standardized weighted low-resolution electromagnetic tomography* (swLORETA), to investigate the neural mechanism subserving the orthographic processing of symbols in language and music. While much is known about word processing, the current literature remains inconclusive regarding music reading, as its mechanisms appear to be left lateralized in some cases (as suggested by music-alexia clinical case reports) and either right-sided or bilateral in others, depending on the study and the methodology used.

**Methods:**

In this study, 90 right-handed participants with varying musical abilities and sexes performed an attentional selection task that involved the recognition of target letters and musical notes, while their EEG signals were recorded from 128 sites.

**Results:**

The occipito/temporal N170 component of ERPs (170–210 ms) was found strictly left-sided during letter selection and bilateral (with a right-hemispheric tendency) during note selection. Source reconstruction data indicated the preponderant engagement of the right posterior fusiform gyrus (BA19) for processing musical notes. Also involved were other brain regions belonging to the word reading circuit, including the left-sided *visual word form area* (VWFA) and *frontal eye-fields* (FEFs).

**Discussion:**

This finding provides an explanation for the infrequent appearance of musical alexia cases (previously observed only in patients with left hemispheric lesions). It also suggests how musical literacy could be a rehabilitative and preventive factor for dyslexia, by promoting neuroplasticity and bilaterality in the reading areas.

## Introduction

Knowledge of orthographic mechanisms for recognizing alphabetic characters, such as letters, is highly developed. Conversely, visual mechanisms for reading and recognizing musical notation exhibit more ambiguity and inconsistency. Many of the inconsistencies relate to the exact location of the mechanism, which should be in the left hemisphere based on the limited data from music alexia. However, numerous studies have proposed a right posterior region that is highly responsive to notation. In this study, we have attempted to possibly identify the electromagnetic sources of notation processing (the *visual note form area*, VNFA) by testing a very large group of participants, including professional musicians, but also people with elementary musical education. It is known that the *Visual Word Form Area* (VWFA), located in the medial portion of the left fusiform gyrus, plays a key role in letter and word recognition, being more responsive to strings of letters rather than other symbols (Kuriki et al., 1998; Cohen et al., 2000, 2003; Garrett et al., 2000; Polk et al., 2002; Flowers et al., 2004; Pernet et al., 2005; Vigneau et al., 2005; Kronbichler et al., 2007).

The development and specialization of this area for recognition of written words enables rapid reading, as it increases the perceptual ability of words, making it sensitive to recurrent features of the writing system (McCandliss et al., 2003). Although the VWFA processes structurally invariant representations of visually presented words as abstract letter sequences, independent of size, style, and character (Dehaene et al., 2002), several studies have shown that the regions of the left fusiform gyrus, corresponding to the VWFA, are sensitive to sublexical properties of words such as their frequency of use and orthographic familiarity (Mechelli et al., 2003; Kronbichler et al., 2004; Devlin et al., 2006; Bruno et al., 2008). Indeed, data have shown higher activations for words than strings of letters and for words with high- than low-frequency of use (e.g., Proverbio et al., 2008).

The precise localization of VWFA in the left occipitotemporal sulcus has been explained by its anatomical proximity to the left perisylvian area, which is deputed to the processing of spoken language; in this context, the VWFA could play the role of providing direct connections between the visual and linguistic areas, and in particular between the ventral visual recognition system and the perisylvian areas of language (Bouhali et al., 2014). The early study by Cohen et al. (2000) found a left lateralization of hemispheric activation in control subjects, regardless of the visual field in which the stimulus was presented. The role of the left occipito-temporal area in word reading was later supported by Devlin et al. (2006), who identified its specific components in the posterior occipito-temporal sulcus and the inferior ventral fusiform and temporal gyrus. The results found an activation of the left posterior fusiform gyrus in both word and pseudoword reading, with peak activation in response to words located in the left occipito-temporal sulcus, and extending to the convexity of the posterior fusiform gyrus and the inferior temporal gyrus. In addition, with regard to the left fusiform gyrus, the more posterior portion would appear to be more responsive to single letters, while the anterior portion would activate more extensively in response to letter strings (James et al., 2005). Thesen et al. (2012) through an fMRI study found that the selective activation for letters in the left posterior fusiform gyrus was prior to more anterior selective activation for words.

The N170 ERP component is the electromagnetic manifestation of VWFA activation, with its negative peak exhibiting the highest amplitude to letter/words than other objects at ~170 ms in the left occipito/temporal region (Bentin et al., 1999; Rossion et al., 2003; Proverbio et al., 2008). The N170 component has also been shown to be sensitive to musical notation, being larger to target than non-target notes at bilateral occipito/temporal sites, in selective attention tasks requiring visual recognition and response to tones of different pitch (Proverbio et al., 2013).

As for the neural mechanism subserving the ability to read musical notation, the pioneeristic study by Sergent et al. (1992) found that reading musical notations and translating these notations into musical gestures resulted in the activation of cortical areas distinct from, but adjacent to, those devoted to orthographic reading. Reading a musical score (without listening or playing) was associated with the activation of bilateral extra-striate visual areas (BA18). However, the left occipito-parietal junction (BA19), rather than the left lingual gyrus (LG) and fusiform gyrus (FG), was activated by musical notation. This was interpreted as a reflection of the role of the dorsal visual system in spatial processing (pentagram, spatial position of notes). In an ERP study conducted with control subjects and musicians, which included notation reading and a selective attention task with notes vs. letters, we discovered that there exists only a partial overlap between reading words and reading notes (Proverbio et al., 2013). In musicians, musical notation recognition strongly activated the left FG (BA27), but also the right inferior occipital gyrus (BA18) and the right FG, BA37). Similarly, Nakada et al. (1998) identified the right transverse occipital sulcus as being more active when reading musical scores compared to reading English or Japanese texts, which activated the VWFA. Additionally, Schön et al. (2001) emphasized the role of the right occipitotemporal junction in music reading, drawing a comparison to word reading. This finding was explained in light of the hypothesis that note pitch is processed with regards spatial position within the pentagram (a right hemispheric function), while words and, more in general, alphanumeric characters, are encoded in relation to their shape. Of course, other musical accidentals such as flats, sharps, pauses, and duration indications are also recognized based on their shape rather than their position. Schön et al. (2001) therefore suggested that the right occipitotemporal junction might represent the musical functional homolog of the VWFA for written words. However, there is not a clear agreement in the literature about the role of right visual areas in notation processing. Mongelli et al. (2017) used fMRI to assess activations induced by musical notation in professional musicians and naive controls, comparing them to activations by written words and other classes of objects. They found that both words and music activated the left occipitotemporal cortex (VWFA), but music-related activations in the left occipitotemporal cortex peaked posterior and lateral to word-related activations. In addition, a bilateral network including the right occipito/temporal cortex was more connected during music than word processing. In another study, Lu et al. (2019), through magnetoencephalography, compared the activations of brain areas during silent word and music reading in professional musicians and found an activation of left sided regions in both music and speech tasks. In addition, it was found the bilateral activation of the superior parietal cortex, which was interpreted as reflecting encoding and visuomotor transformation (as also found by Meister et al., 2004; Proverbio et al., 2014). The parietal cortex was also observed to be active in an fMRI study by Stewart et al. (2003), in which a group of non-musicians underwent training to learn to play the piano and read music for 15 weeks. Post-training scans revealed increased activation of the right superior parietal cortex and supramarginal gyrus, consistent with other imaging studies (Sergent et al., 1992; Schön et al., 2001; Roux et al., 2007). Finally, Wong and Gauthier (2010) tested the ability to selectively attend to part of a four-note sequence in music reading experts and novices in a same/different task and found a correlation between right FG activation and holistic strategy.

All in all, while its seems that reading musical activates right posterior areas to some extent, there is not complete agreement in the literature about the precise network involved in the orthographic stage of music processing (150–200 ms), that from study to study would involve the transverse occipital sulcus, the occipital gyrus, the inferior occipital gyrus, the superior occipital gyrus, the occipito/temporal junction, the FG, the superior parietal and supramarginal cortices. These differences may be due to the different tasks or methodological constraints used to test reading of musical notation: experimental paradigms range from reading while playing, reading in silence, reading at first sight, attentional selection of notes, reading for learning to play, etc. For example, Lu et al. (2019) used MEG to compare reading of musical notation vs. English letters in professional musicians. Both note and letter reading tasks required translation to phonological codes and activated left hemisphere language areas and bilateral parietal cortex. In addition, the authors measured a laterality index to determine which of the two hemispheres was more active during a 1–letter sounding and a 1-note reading tasks, and found that in about half of the participants the left fusiform gyrus was more activated than the right fusiform gyrus (and vice versa) in the 150–200 ms time window: these results do not provide a convincing pattern of results.

Overall, some music reading studies indicate greater visual activation on the right side (Nakada et al., 1998; Li et al., 2017; Lu et al., 2021), whereas others suggest a more bilateral distribution (Proverbio et al., 2013) or smaller activation on the right side compared to the left (Mongelli et al., 2017). One potential theoretical problem with the neuroimaging literature is that music alexia, a relatively rare disorder that consists of an inability to recognize musical notes, is almost invariably linked to left-hemispheric lesions. Indeed, Peretz and Zatorre (2005) assumed that music sight-reading would involve left occipito/temporal areas, whose lesion would lead to music alexia (e.g., Schön et al., 2001).

There are three variants of music alexia that have been delineated over the years: difficulty in reading individual notes (Fasanaro et al., 1990; Cappelletti et al., 2000), misreading the pitch of notes rather than the rhythm (Brust, 1980), and inability to recognize both rhythm and correct pitch (Brust, 1980; Midorikawa et al., 2003). In all quoted cases, and in others, music alexia was consistently associated with a left hemispheric lesion; more specifically this was observed in the left temporoparieto-occipital region in Fasanaro et al. (1990), in the left posterior temporal lobe (along with a small right occipitotemporal lesion) in Cappelletti et al. (2000), in the left temporal lobe in Brust (1980), in the left superior temporal gyrus in Midorikawa et al. (2003), in the left angular gyrus in Kawamura et al. (2000), and in the left ventral temporo-occipital cortex in Starrfelt and Wong (2017). Again, one of the earliest cases of musical alexia reported by Horikoshi et al. (1997) regarded the case of a 26-year-old pianist who, due to an intracerebral hematoma in the left occipital lobe, experienced difficulty in reading music, especially in relation to the pitch of notes, while maintaining preserved rhythm reading, auditory recognition, and playing ability.

Note and word reading exhibit related processing and production processes, although functionally the areas involved are close but, for the most part, not overlapping. This, in fact, provides an explanation with respect to the possibility of finding clinical case descriptions that report associations or dissociations between language disorders and musical disorders. Despite cases of music alexia being associated with damage to the left occipito/temporal or occipito/parietal cortex, recent neurometabolic and electromagnetic studies have demonstrated the crucial role of the right visual cortex in reading notes and playing music scores. We aim to clarify the matter by gauging participants' capacity to discern and identify written words and musical notes. This will be achieved by observing an unprecedentedly large sample of strictly right-handed participants and comparing their ability to discern and identify written words and notes. In order to measure the neurological response to both stimuli, we'll be considering N170 responses for electromagnetic manifestation. These responses will be studied through brain source reconstruction for participants with varying degrees of musical experience.

## Methods

### Participants

Ninety healthy, right-handed post-graduate students from the Milan area participated in the experiment. EEG data from four participants were disregarded due to an abundance of EEG artifacts, and two participants were disqualified due to amplifier blocking. The final sample consisted of 36 male and 48 female participants, with a mean age of 25.6 years (SD = 6.3). Half of the participants were professional musicians with a Conservatory degree and typically read music scores in the Violin and/or Bass clefs (44 musicians), whereas the other half had studied music for ~3 years in high school (40 non-musicians). They were matched for socio-cultural status, and were all native Italian speakers. Their lateral preference was assessed through the administration of the Edinburgh Inventory (Oldfield, 1971) whose scale ranges from −1 (indicating left-handedness) to +1 (indicating right-handedness). Musicians' average laterality score was 0.714 (standard deviation = 1.18), compared to non-musicians' average score of 0.80 (standard deviation = 1.145), indicating that all participants were fully right-handed (average score = 0.76). Inclusion criteria were having never suffered from psychiatric or neurologic disorders, not being currently assuming drugs or narcoleptics, or being epilepsy-predisposed. All participants had normal or glass-corrected vision and hearing. No participant suffered or ever suffered from learning or reading disorder (e.g., developmental dyslexia, alexia, autism, ADHD, etc...). All participants provided written informed consent and were unaware of the research purpose. The study lasted approximately 3 h, encompassing breaks, training, and administering the questionnaire. Participants voluntarily offered their participation or received academic credits. The Ethics Committee of University of Milano-Bicocca (protocol number RM-2021-370) approved the project.

### Stimulus and procedure

The research included 300 Italian words and 300 music bars with different lengths and complexities. These were presented randomly on a computer screen. The stimuli used were identical to those utilized in an earlier ERP study (Proverbio et al., 2013). The words were typed in capital letters and varied in length from 4 to 10 letters, while the music bars were 4–8 notes in length. Two experimental conditions were used: a note recognition task and a letter recognition task. They consisted in pressing a key at the sight of a specific letter contained in a word, or note contained in a musical measure, depending on the experimental condition, while ignoring non-target letters or notes. The participants completed one task in the first half, and the other in the second half of the experiment, with stimulus presentation lasting 1,600 ms and an ISI of 1,000 to 1,200 ms. The stimuli were matched for duration, frequency of use, and target/non-target categories. For the note recognition task, the targets were “mi,” “fa,” “sol,” “la,” and “si” of the middle piano octave, while the orthographic task used the letters B, G, L, M, and S. Participants received verbal and visual cues before each trial, and responded by pressing a joystick button with their index finger. Stimuli were randomly presented in 12 runs of 50, with halves of targets and non-targets. The two experimental sessions were preceded by two training sequences, and a fixation point was used to minimize movement. The response hand and presentation order were both counterbalanced across participants.

### EEG recordings

EEG data were recorded using a 128-electrode cap, at a sampling rate of 512 Hz. hEOG and vEOG eye movements were recorded and linked mastoids were used as a reference lead. Electrode impedance was kept below 5 KOhm and artifact rejection methods removed contaminated EEG segments. A computerized criterion for rejecting artifacts was a peak-to-peak amplitude exceeding 50 μV. Evoked response potentials (ERPs) were then averaged off-line and a band-pass 0.16–30 Hz filter was applied to them. ERPs were averaged offline with a time epoch of 1,500 ms. N170 orthographic response was identified and measured in the time window and scalp location where it reached its maximum amplitude, and according to previous literature (e.g., Proverbio et al., 2013; Cnudde et al., 2021). N170 mean area amplitude of the N170 was quantified at occipito-temporal sites (PPO9h-PPO10h) in between 170 and 210 ms in response to notes and words, in musicians and controls, and across the different attentional conditions. Behavioral data (response times in ms) were additionally recorded.

Individual N170 amplitude values were subjected to repeated measures ANOVA. Factors of variance were: 1 between-group factor “group” (musicians vs. controls) plus 3 within-group factors: “stimulus type” (notes vs. words), “target” (non-targets, targets); “hemisphere” (left, right). A further repeated-measure ANOVA was performed on response times (RTs) data. Between group factor was “group” (musicians vs. controls). Within-group factors were: “stimulus type” (note vs. word), “target” (non-target, target), and “response hand” (left, right).

Tukey *post-hoc* comparisons were carried out to test differences among means. The effect size for the statistically significant factors was estimated using partial Eta Squared ($\eta_{p}^{2}$) and the Greenhouse-Geisser correction was applied to account for non-sphericity of the data.

### Source reconstruction

In order to identify the intracranial sources explaining the surface electrical potentials Standardized low-resolution electromagnetic tomography (sLORETA; Pascual-Marqui, 2002) was performed on ERP voltages. In particular, LORETA was applied to mean voltages recorded in the 170–190 ms time range, and corresponding to N170 response. LORETA is a discrete linear solution to the inverse EEG problem, and it corresponds to the 3D distribution of neural electric activity that maximizes similarity (i.e., maximizes synchronization) in terms of orientation and strength between neighboring neuronal populations (represented by adjacent voxels). In this study, an improved version of standardized weighted low-resolution brain electromagnetic tomography was used (swLORETA, Palmero-Soler et al., 2007), which incorporates a singular value decomposition-based lead field weighting method. The source space properties included: localization within the gray matter; a grid spacing of five points (the distance between 2 calculation points) and an estimated signal-to-noise ratio (SNR) of 3, which defines the regularization (higher values indicating less regularization and therefore less blurred results). Using a value of 3–4 for SNR computation in Tikhonov's regularization results in superior accuracy for all assessed inverse problems. swLORETA was performed on the grand-averaged data to identify statistically significant electromagnetic dipoles (*p* < 0.05) in which larger magnitudes correlated with more significant activation. The data were automatically re-referenced to the average reference as part of the LORETA analysis. A realistic boundary element model (BEM) was derived from a T1-weighted 3D MRI dataset through segmentation of the brain tissue. This BEM model consisted of a homogeneous compartment comprising 3,446 vertices and 6,888 triangles. Advanced source analysis (ASA) employs a realistic head model of three layers (scalp, skull, and brain) created using the boundary element model. This realistic head model comprises a set of irregularly shaped boundaries and the conductivity values for the compartments between them. Each boundary is represented by a series of interconnected points, forming plane triangles to create an approximation. The triangulation leads to a more or less evenly distributed mesh of triangles as a function of the chosen grid value. A smaller value for the grid spacing results in finer meshes and vice versa. With the aforementioned realistic head model of three layers, the segmentation is assumed to include current generators of brain volume, including both gray and white matter. Scalp, skull, and brain region conductivities were assumed to be 0.33, 0.0042, and 0.33, respectively. The source reconstruction solutions were projected onto the 3D MRI of the Collins brain, which was supplied by the Montreal Neurological Institute. The probabilities of source activation based on Fisher's *F* test were provided for each independent EEG source, whose values are indicated in a “unit” scale in *nA* (the larger the value, the more significant the activation). It should be noted, however, that the spatial resolution of swLORETA is somewhat limited compared to other neuroimaging techniques like MEG or fMRI. Both the head model's segmentation and generation were conducted through the use of Advanced Neuro Technology, a software program developed by ASA (Zanow and Knösche, 2004).

## Results

### Behavioral data

**Figure 1:**
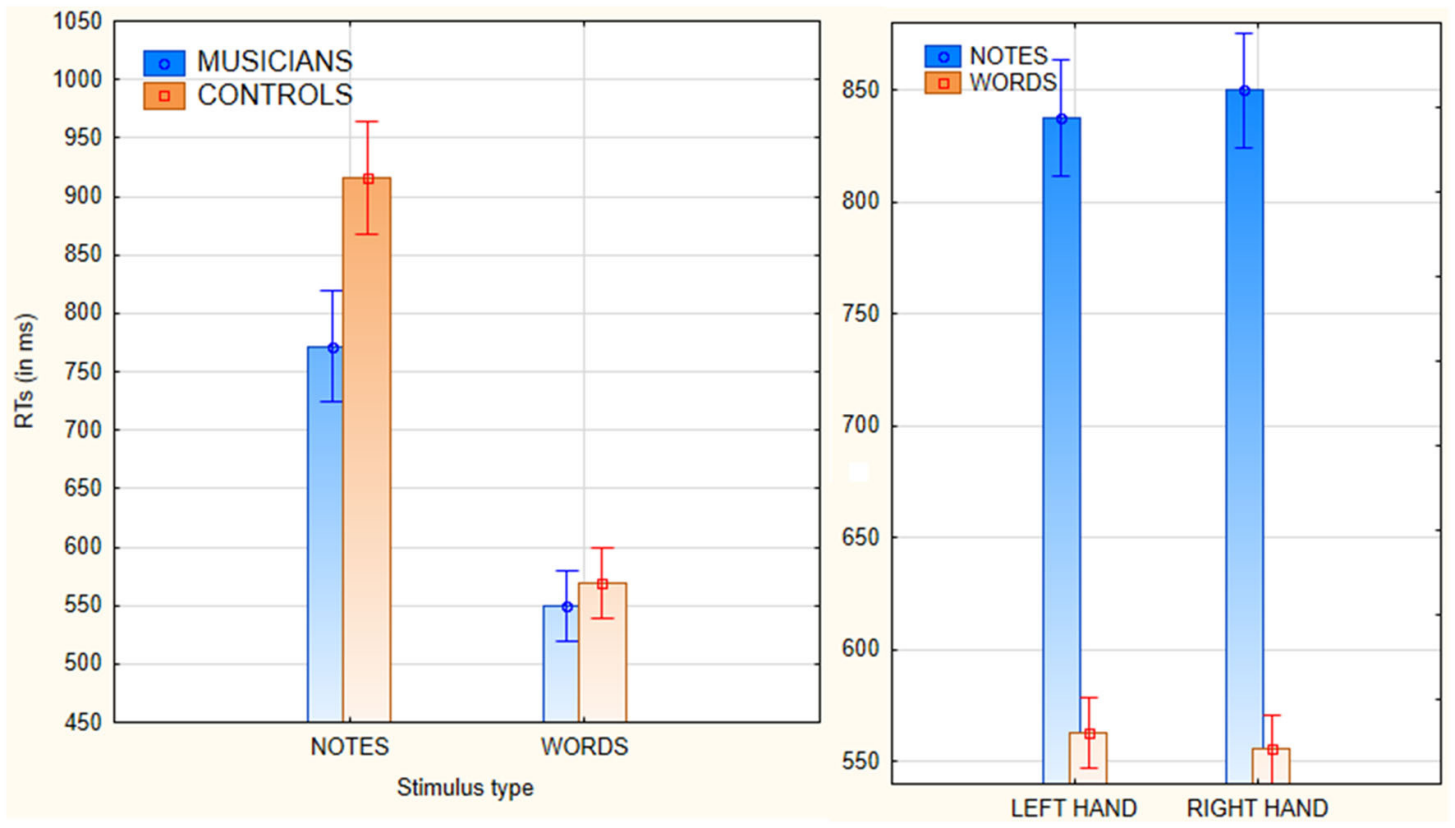
**(Left)** Mean reaction times (with standard errors) recorded in response to the two types of stimuli in musicians and non-musicians. **(Right)** Mean reaction times (with standard errors) performed with the left vs. the right hand, in response to stimuli of the two types.

The ANOVA yielded the significance of group [*F*_(1, 82)_ = 28.7, *p* < 0.00001; $\eta_{\text{p}}^{2}$ = 0.26], with faster RTs in musicians than controls (Figure 1, left). Also significant was the factor stimulus type [*F*_(1, 82)_ = 785, *p* < 0.00001; $\eta_{\text{p}}^{2}$ = 0.91], with faster responses to words than notes. The significant interaction of stimulus type x group [*F*_(1, 82)_ = 40.35, *p* < 0.00001; $\eta_{\text{p}}^{2}$ = 0.33], and relative *post-hoc* comparisons, showed faster RTs to words than to notes in both groups, faster RTs to notes in musicians than in controls (*p* < 0.00001), but not significantly faster RTs to words in musicians (550 ms, SE = 10.5) than in controls (570.3 ms, SE = 10.8), as can be seen in Figure 1, left. The further interaction of response hand x stimulus type [*F*_(1, 82)_ = 4.1, *p* < 0.05; $\eta_{\text{p}}^{2}$ = 0.04] showed faster (*p* < 0.00001) RTs to words with the right hand (Figure 1, right), but a non-significant trend (*p* = 0.1) for faster RTs to notes with the left than with the right hand.

### Electrophysiological data

**Figure 2:**
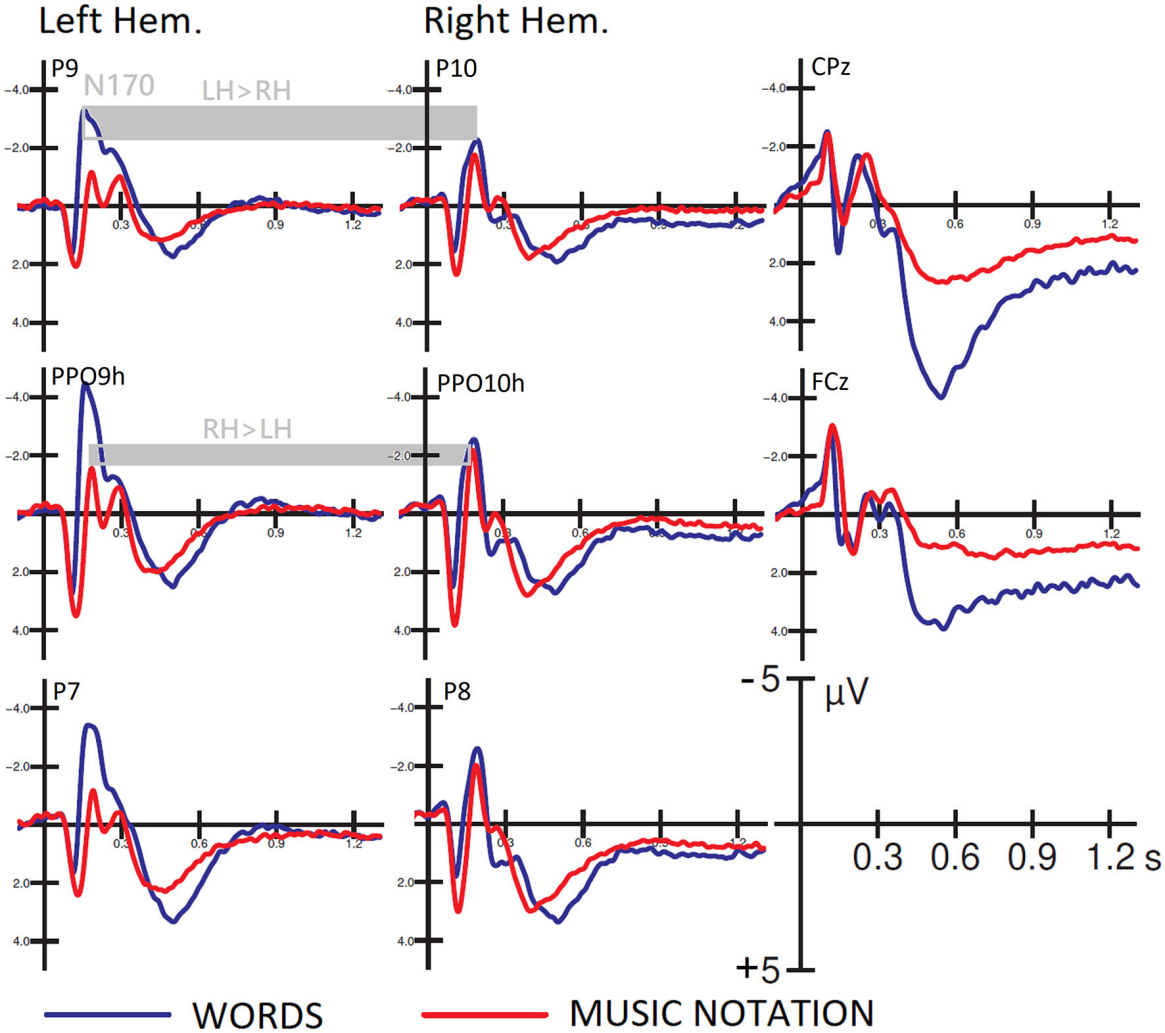
Grand-average ERP waveforms recorded at left and right posterior temporal and occipito/temporal sites, and midline cento/parietal and fronto/central sites as a function of stimulus type. Hemispheric asymmetries in favor of the left hemisphere for word processing and the right hemisphere for note processing are highlighted.

Figure 2 shows the grand-average ERP waveforms recorded in response to the two types of stimuli over the left and right occipito-temporal cortex, where the N170 showed its maximum distribution, and over the midline centro-parietal and frontal areas. It can be observed the presence of consistent hemispheric asymmetries, dependent on stimulus type, which were statistically analyzed.

**Figure 3:**
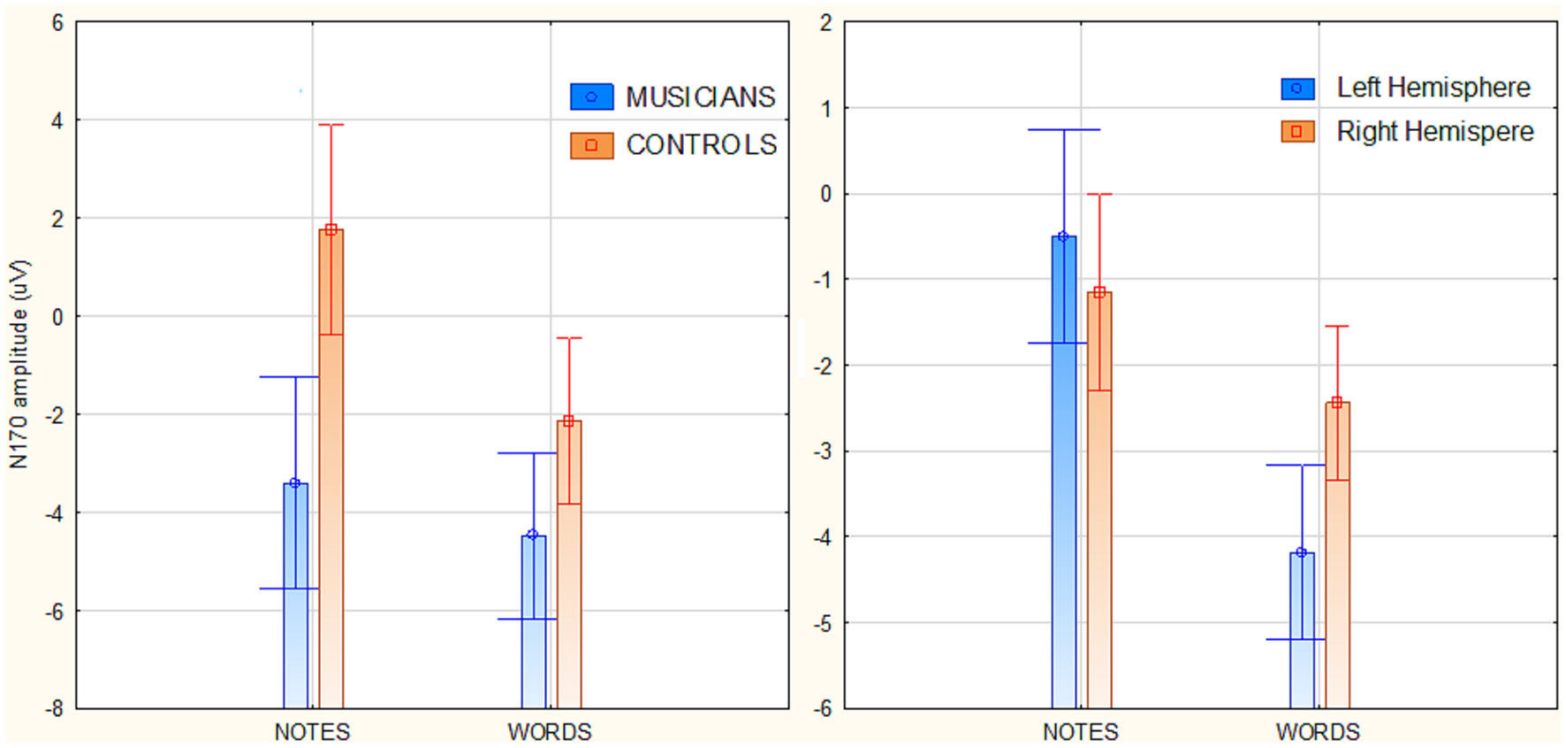
**(Left)** Mean amplitude values of the occipito/temporal N170 response (170–210 ms) as a function of participants' musical education and stimulus type. **(Right)** Mean amplitude values of the occipito/temporal N170 response as a function of stimulus type and cerebral hemisphere.

**Figure 4:**
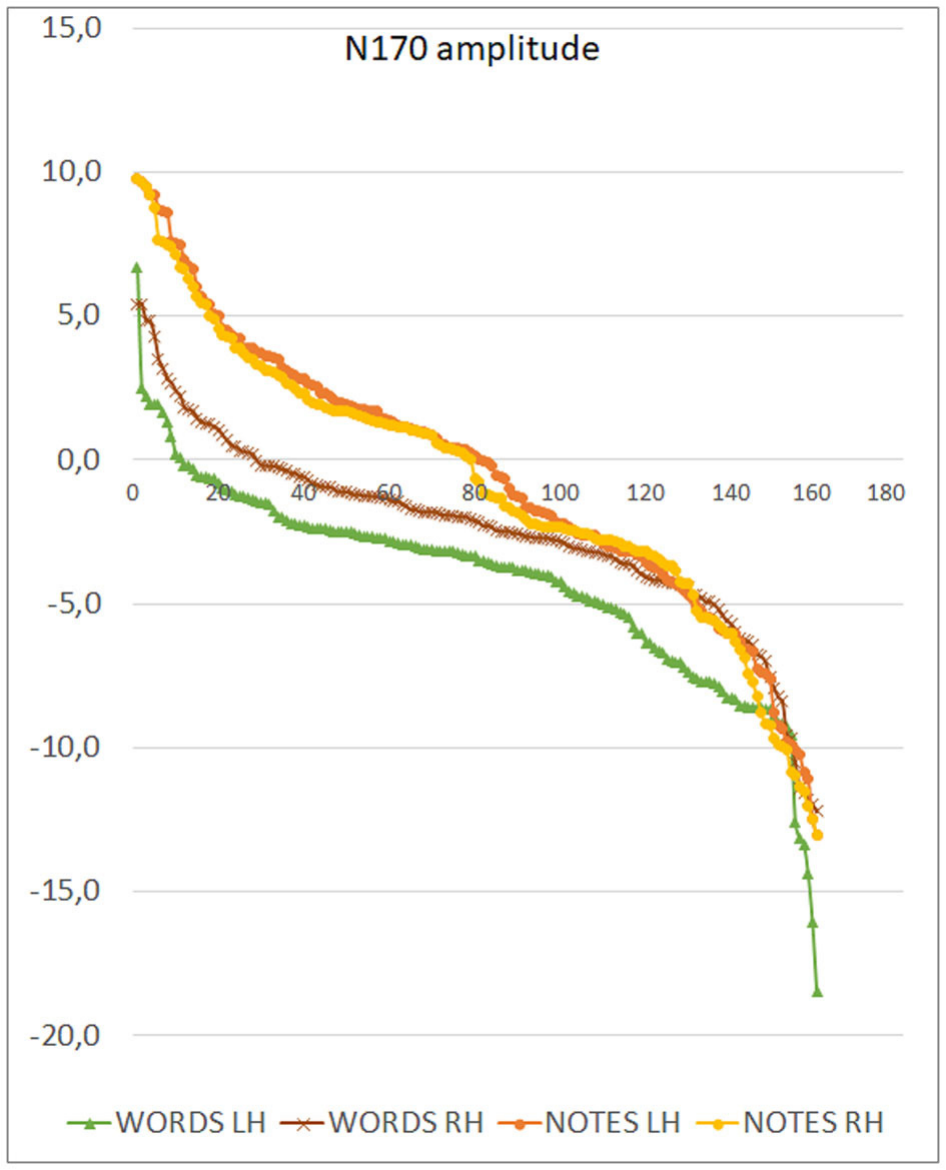
Distribution of individual values of N170 amplitudes recorded in individual participants as a function of stimulus type and cerebral hemisphere. Note the macroscopic left lateralization of the N170 in response to words and the more subtle inverse pattern (consistent across participants) for notes.

The ANOVA yielded the significance of group [*F*_(1, 82)_ = 40.23, *p* < 0.001; $\eta_{p}^{2}$ = 0.33], with much larger N170 responses recorded in musicians than in controls (see Figure 3 left, for mean and SE values). Also significant was the stimulus type factor [*F*_(1, 82)_ = 61, *p* < 0.001; ε = 1; $\eta_{p}^{2}$ = 0.44], with larger N170 to words than to notes. The interaction of group x stimulus type [*F*_(1, 82)_ = 23.78, *p* < 0.001; ε= 1; $\eta_{p}^{2}$ = 0.74], and relative *post-hoc* comparisons, showed significantly larger N170 to words than to notes in both groups of controls (*p* < 0.001) and musicians (*p* < 0.039). Furthermore, N170 was markedly larger in musicians than controls in response to both notation (*p* < 0.001), and words (*p* < 0.001). The ANOVA also yielded the significance of hemisphere factor [*F*_(1, 82)_ = 4.5, *p* < 0.035; ε = 1; $\eta_{p}^{2}$ = 0.05] with larger N170 amplitudes over the left than the right hemisphere (Figure 2, right). The further interaction of stimulus type x hemisphere [*F*_(1, 82)_ = 24.25, *p* < 0.001; ε = 1; $\eta_{p}^{2}$ = 0.23], and relative *post-hoc* comparisons, showed that while N170 was larger over the left than right hemisphere to words (*p* < 0.001), it tended to be larger over the right than left hemisphere to notes (*p* < 0.07) as shown in Figure 3 (right) and in the scatterplot distribution of individual values displayed in Figure 4.

**Figure 5:**
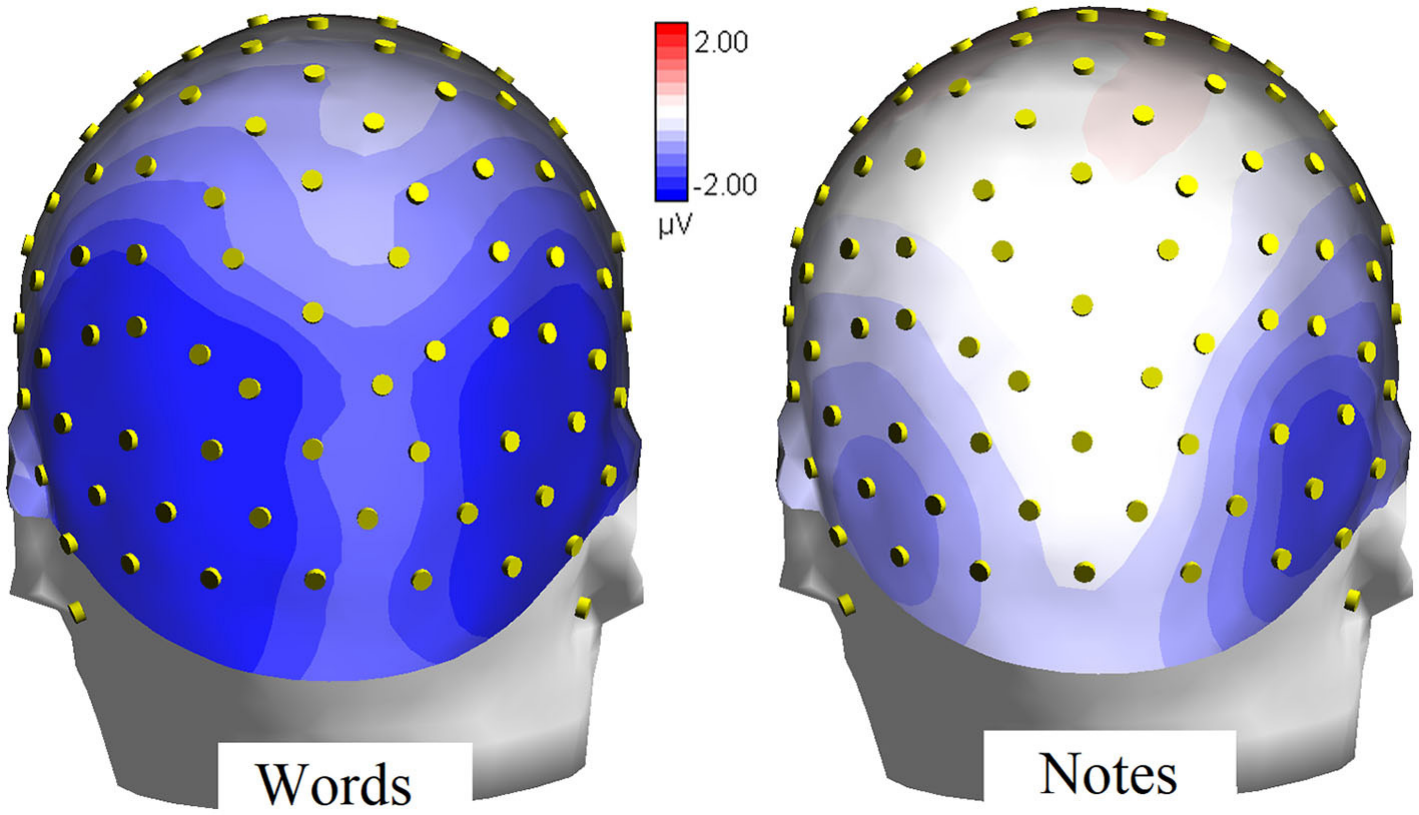
Iso-Color topographical maps (back view) of N170 voltage recorded in the 170–210 ms time window during processing of word and musical notation.

**Figure 6:**
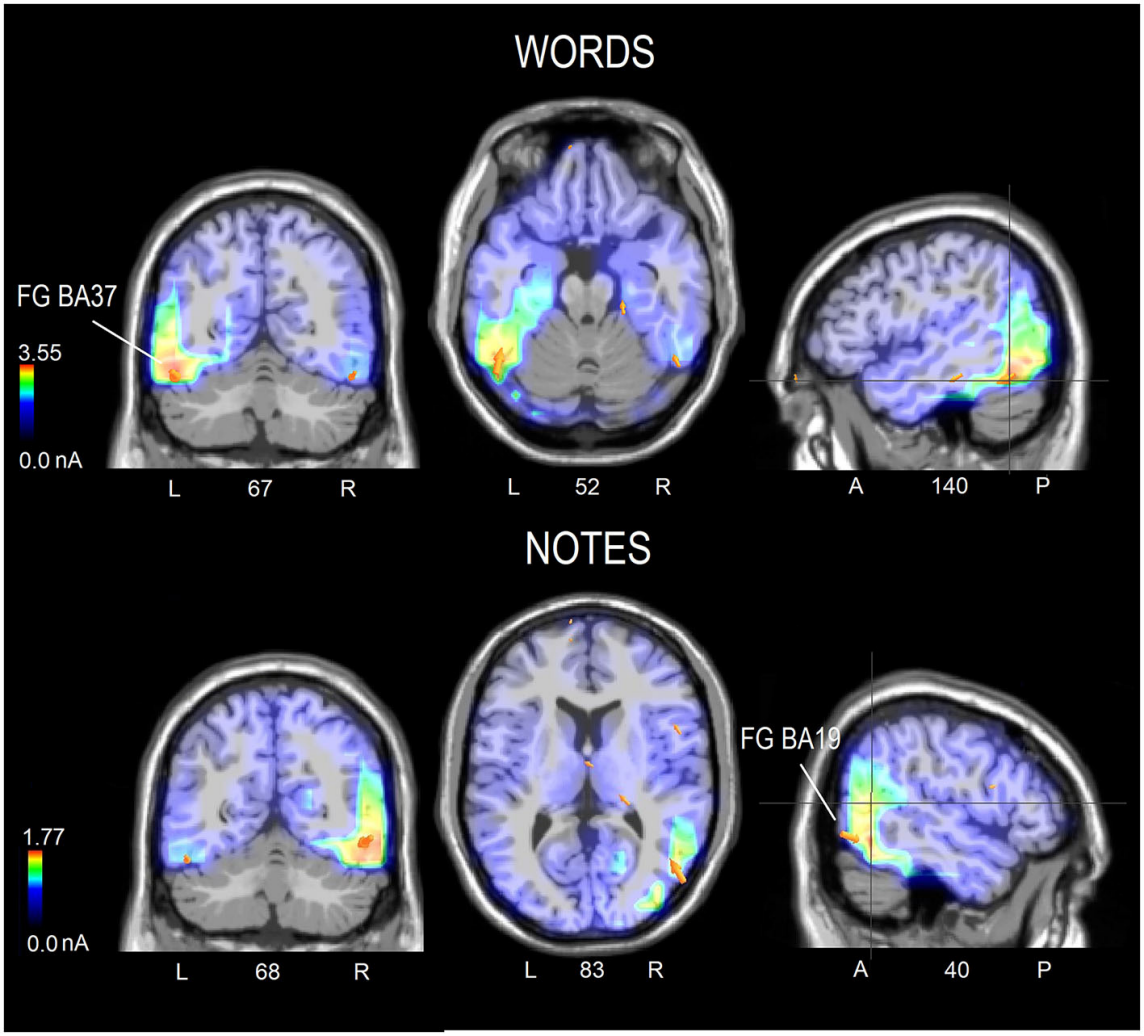
Coronal, axial and sagittal brain sections showing the location and strength of electromagnetic dipoles explaining the surface voltage of N170 response (170–210 ms) in response to words (upper row) or notes (lower row) in the whole sample of participants. The selected sections were centered on the sources of the fusiform gyrus in both cases. The electromagnetic dipoles are shown as arrows and indicate the position, orientation and magnitude of dipole modeling solution.

Figure 5 displays the topographical distribution of N170 voltage response to words and notes within the 170–210 ms latency range. The left-sided distribution of the orthographic response during word processing is evident, as well as the right-sided distribution during musical notation processing. An inversion solution was applied to N170 mean voltages recorded in the 170–190 ms time range, in order to identify the inner sources of surface potentials. Table 1 provides a comprehensive list of active electromagnetic dipoles separately for word and notation processing, while the corresponding neuroimages are shown in Figure 6. During word reading, the most active source was undoubtedly the left fusiform gyrus (BA37), with a lesser activation of the contralateral counterpart. During note reading, the most active source was the right fusiform gyrus (BA19), with smaller amplitudes in the left FG BA37 and in the left inferior temporal gyrus (BA20).

**Table 1:** List of active electromagnetic dipoles (along with their Talairach coordinates and relative Brodmann areas) explaining the scalp-recorded potentials measured in the 170–210 ms time windows in response to words and notes in the whole sample of participants.

**Words**							
**Magn**.	**X [mm]**	**Y [mm]**	**Z [mm]**	**H**	**Lobe**	**Gyrus**	**BA**
1.35	−48.5	−55.0	−17.6	L	T	Fusiform	37
1.20	50.8	−55.0	−17.6	R	T	Fusiform	37
1.19	21.2	−24.5	−15.5	R	Limbic	Parahippocampal	35
1.16	40.9	−87.3	−4.9	R	O	Inferior Occipital	18
1.13	1.5	−20.3	26.8	R	Limbic	Cingulate Gyrus	23
1.11	−8.5	57.3	−9.0	L	F	Superior Frontal	10
0.97	−38.5	−21.0	35.7	L	P	Postcentral	3
0.77	31.0	−7.0	46.3	R	F	Medial Frontal	6
0.49	21.1	52,4	33.7	R	F	Superior Frontal Gyrus	9
0.35	1.5	40.5	50.7	R	F	Superior Frontal Gyrus	8
**Notes**							
1.18	50.8	−66.1	−10.9	R	T	Fusiform	19
1.11	−48.5	−55.0	−17.6	L	T	Fusiform	37
0.86	−38.5	−15.3	−29.6	L	T	Inferior Temporal	20
0.83	50.8	3.3	20.5	R	F	Inferior Frontal	44
0.76	1.5	−13.0	27.7	R	Limbic	Cingulate	23
0.43	−8.5	64.4	16.8	L	F	Superior Frontal	10
0.41	−8.5	57.3	−9.0	L	F	Superior Frontal	10
0.40	−8.5	52.4	33.7	L	F	Superior Frontal	9
0.37	1.5	40.5	50.7	R	F	Superior Frontal	8

The strength of the electromagnetic dipoles (magnitude) is expressed in nA (nanoampere).

Magn., magnitude; H, hemisphere; BA, Brodmann areas.

Common areas of activation during reading of words and music were observed. They were the superior frontal gyrus (BA8), devoted to attention and gaze shifting; the right cingulate cortex (BA23) devoted to non-target suppression and response selection; and the superior frontal gyri (BA9 and 10), involved in working memory and selective attentional control. Interestingly, some sources were uniquely active during word reading, namely the parahippocampal area (BA35), which is active during reading tasks, the right inferior occipital gyrus (BA18) and the left parietal area (BA3), which are involved in letter shape extraction during reading. On the other hand, other sources were uniquely active during reading music, namely the left inferior temporal gyrus (BA20), involved in shape recognition and selection, and the right inferior frontal gyrus (BA44), involved in encoding musical metrics and tempo.

## Discussion

This study aimed to investigate the neural mechanisms involved in visually recognizing notes and letters (in the context of music bars and words) in a large sample of right-handed individuals with varying levels of musical familiarity. We also aimed to address the seeming inconsistency of music alexia having a left hemispheric basis, despite neuroimaging data suggesting a privileged role or involvement (depending on the study) of the right occipital/temporal region in music reading. Overall, the whole sample here considered showed a right hand/left hemisphere preference for responding to target letters, and a bilaterality (with a tendency in favor of the left hand/right hemisphere) for responding to target notes. The N170 component of ERPs showed a predominantly left-sided response to words, consistent with prior electrophysiological literature (Bentin et al., 1999; Rossion et al., 2003; Proverbio et al., 2008). In contrast, the N170 response was bilateral and tended to be larger over the right hemisphere in response to music stimuli, as shown in the topographical maps of surface voltage plotted across all electrodes. LORETA source reconstruction showed how word encoding during letter selection strongly activated the left fusiform gyrus (BA37) corresponding to the putative VWFA (Bentin et al., 1999; Rossion et al., 2003; Proverbio et al., 2008; Selpien et al., 2015; Dehaene-Lambertz et al., 2018). The left mid-fusiform cortex is thought to specialize in letter and word visual recognition with the acquisition of literacy (Cohen et al., 2002; McCandliss et al., 2003; Cohen and Dehaene, 2004; Szwed et al., 2011; Glezer et al., 2015). The left lateralization (as opposed to the right one) can be attributed to this region's inclination to analyze local elements, such as high spatial frequencies and details. This is in contrast to the holistic characteristics of neurons in the homologous right visual area, i.e., the fusiform face area, which prefer to recognize holistic patterns such as face schemata (Davies-Thompson et al., 2016; Takamiya et al., 2020).

The activity observed in the right fusiform gyrus during letter selection in this study could be attributed to the fact that around half of the participants were musicians. It is recognized that musicianship may enhance the development of the right reading area for both notation and language reading, potentially explaining this phenomenon (Proverbio et al., 2013, 2024). Other regions that were uniquely active during word reading included the parahippocampal gyrus (BA35), which is strongly connected to the VWFA (van der Mark et al., 2011; Fan et al., 2014) and involved in reading (Sefcikova et al., 2022), as well as the right inferior occipital gyrus (BA18) and left parietal area (BA3), which are thought to be involved in letter shape extraction during reading (Corbetta and Shulman, 2002; Zhang et al., 2013). In addition, other areas were found to be commonly active during reading of words and music. They were the superior frontal gyrus (BA8), also known as *frontal eye-fields* (FEF) controlling attentional and gaze shifting (Japee et al., 2015; Wang et al., 2019); the right cingulate cortex (BA23), involved in non-target suppression and response selection (Braver et al., 2001); and the superior frontal gyrus (BA9 and BA10), involved in working memory (Yee et al., 2010) and selective attentional control (Proverbio et al., 2009; Szczepanski et al., 2013).

Note reading activated only partially overlapping circuits largely extended over the right hemisphere. The strongest focus of activation during note reading was the right posterior fusiform gyrus (BA19). This area lies in similar regions or coincides with those described in previous literature on notation reading (Sergent et al., 1992; Nakada et al., 1998; Wong and Gauthier, 2010; Li et al., 2017; Lu et al., 2021). Along with the right FG were also strongly active during note selection the left fusiform gyrus (BA37) and the left inferior temporal gyrus (BA20), possibly involved in shape recognition of notation symbols, such those indicating key, duration, sharps, flats, pauses, agogic or tonal articulation. On the other hand, right hemispheric visual areas, such as the right fusiform gyrus, would preferentially process holistic information and be activated during selective attention to global configurations (Proverbio et al., 1998). In fact, the activation of the fusiform face area (FFA) during face processing is mostly right-sided (Kanwisher et al., 1997), especially in males. Holistic face processing has been consistently demonstrated across various paradigms, such as the part-whole (Tanaka and Simonyi, 2016) and composite paradigms (Rossion, 2013). We believe that the right fusiform gyrus cell's broader receptive field could potentially learn to identify notes' pitch across the rows of the pentagram as a whole, while taking into account the global visuospatial coordinates. Consistently, bilateral fusiform gyrus has shown increased selectivity for single notes in experts (Wong and Gauthier, 2010). Interestingly Wong and Gauthier (2010) measured holistic processing of music sequences using a selective attention task in participants who varied in music reading expertise. The authors found that holistic effects were strategic in novices but relatively automatic in experts. Correlational analyses revealed that individual holistic effects were predicted by both individual music reading ability and neural responses for musical notation in the right fusiform face area (rFFA).

In the present study, other neural sources that were uniquely active during reading music and not words (at this early orthographic stage), were the left inferior temporal gyrus (BA20), involved in shape recognition and selection, and the right inferior frontal gyrus (BA44), involved in encoding musical metrics and tempo (Thaut et al., 2014).

As for the effect of musicianship (not the focus of this investigation) the data showed how musicians (*N* = 44) were faster in detecting letters and notes than Non-Musicians (*N* = 40). This finding correlates with electrophysiological data indicating how N170 was markedly larger in musicians than controls in response to both notation and words. This pattern of results fits with previous investigations showing larger N170 words in musicians than non-musicians (Proverbio et al., 2013, 2024) and providing evidence of enhanced reading skills in musicians (Gordon et al., 2015). Correlational studies conducted in adults have demonstrated that individuals who are musicians possess higher sensitivity to speech rhythm (Marie et al., 2011) and exhibit better skills related to reading (such as phoneme discrimination, as observed by Zuk et al., 2013). Whilst the advantageous effects of musical education may lead to neuroplastic changes that affect phonological processing, awareness and sensitivity of the auditory cortex, the observed effect at N170 level reflects a more proficient orthographic encoding, as N170 is believed to manifest VWFA activity. A study conducted by Proverbio et al. (2024) demonstrated that musical education promotes the development of a right-sided area responsible for coding global and spatial properties of musical notation. This area seems to correspond to the right middle occipital/fusiform gyrus, while in musicians and proficient readers, the right middle and inferior temporal cortices (BA20/BA21) nearby would also be activated during word reading. The activity of this right orthographic area would be correlated with the word reading proficiency as measured in independent reading tests (in syllables per second). Consistently Li and Hsiao (2018) showed that musicians named English words significantly faster than non-musicians when words were presented in the left visual field/right hemisphere (RH) or the center position, suggesting an advantage of RH processing due to music reading experience.

Overall, the bilateralism of notation reading mechanism might possibly explain why music dyslexia is so rare, and usually linked to word dyslexia (Brust, 1980; Fasanaro et al., 1990; Cappelletti et al., 2000; Midorikawa et al., 2003). Indeed, the presence of a double mechanism for coding fine grain details and whole spatial information related to music writing, might allow reorganization and neuroplastic compensations leading to preserved music literacy in case of unilateral lesion. Furthermore, the presence of a bilateral redundant reading mechanism, although differently specialized in the analysis of more detailed vs. spatially distributed patterns, in the left and right hemispheres, respectively, might serve as a protective measure against tackling reading disorders or dyslexia. Indeed, orthographic coding mostly engages the left VWFA in non-musicians or non-bilinguals, and studies have shown an atypical/insufficiency activity of the VWFA in surface dyslexic readers (Wilson et al., 2013; Amora et al., 2022) or in poor readers (Proverbio et al., 2024).

## Data availability statement

The original contributions presented in the study are included in the article/supplementary material, further inquiries can be directed to the corresponding author.

## Ethics statement

The studies involving humans were approved by Ethical Committee of University of Milano-Bicocca, Research Evaluation Committee of the Department of Psychology (CRIP). The studies were conducted in accordance with the local legislation and institutional requirements. The participants provided their written informed consent to participate in this study. Written informed consent was obtained from the individual(s) for the publication of any potentially identifiable images or data included in this article.

## Author contributions

AP: Conceptualization, Data curation, Formal analysis, Investigation, Methodology, Resources, Supervision, Writing – original draft, Writing – review & editing. GA: Data curation, Formal analysis, Investigation, Methodology, Validation, Visualization, Writing – original draft. MP: Data curation, Formal analysis, Investigation, Methodology, Validation, Visualization, Writing – original draft. AZ: Formal analysis, Methodology, Software, Visualization, Writing – original draft. MM: Data curation, Formal analysis, Investigation, Methodology, Writing – original draft.
